# Reinforcement Learning-Based Multi-AUV Adaptive Trajectory Planning for Under-Ice Field Estimation

**DOI:** 10.3390/s18113859

**Published:** 2018-11-09

**Authors:** Chaofeng Wang, Li Wei, Zhaohui Wang, Min Song, Nina Mahmoudian

**Affiliations:** 1 Department of Electrical and Computer Engineering, Michigan Technological University, Houghton, MI 49931, USA; cwang8@mtu.edu (C.W.); liwei@mtu.edu (L.W.); 2 Department of Electrical and Computer Engineering, Stevens Institute of Technology, Hoboken, NJ 07030, USA; msong6@stevens.edu; 3 Department of Mechanical Engineering-Engineering Mechanics, Michigan Technological University, Houghton, MI 49931, USA; ninam@mtu.edu

**Keywords:** underwater communication networks, under-ice exploration, field estimation, AUVs, adaptive trajectory planning, reinforcement learning

## Abstract

This work studies online learning-based trajectory planning for multiple autonomous underwater vehicles (AUVs) to estimate a water parameter field of interest in the under-ice environment. A centralized system is considered, where several fixed access points on the ice layer are introduced as gateways for communications between the AUVs and a remote data fusion center. We model the water parameter field of interest as a Gaussian process with unknown hyper-parameters. The AUV trajectories for sampling are determined on an epoch-by-epoch basis. At the end of each epoch, the access points relay the observed field samples from all the AUVs to the fusion center, which computes the posterior distribution of the field based on the Gaussian process regression and estimates the field hyper-parameters. The optimal trajectories of all the AUVs in the next epoch are determined to maximize a long-term reward that is defined based on the field uncertainty reduction and the AUV mobility cost, subject to the kinematics constraint, the communication constraint and the sensing area constraint. We formulate the adaptive trajectory planning problem as a Markov decision process (MDP). A reinforcement learning-based online learning algorithm is designed to determine the optimal AUV trajectories in a constrained continuous space. Simulation results show that the proposed learning-based trajectory planning algorithm has performance similar to a benchmark method that assumes perfect knowledge of the field hyper-parameters.

## 1. Introduction

Autonomous underwater vehicles (AUVs) are attractive platforms for remote underwater exploration and monitoring, e.g., seafloor mapping [1,2] and under-ice hydrographic observations [3]. The AUV trajectories can be determined prior to the deployment or adjusted online based on recent observations. Given the high deployment cost of AUVs, adaptive trajectory planning is desirable for the collection of the “best” data over scalar or vector fields that vary in a range of spatial and temporal scales [4,5,6,7]. This work studies the online adaptive trajectory planning of multiple AUVs in the under-ice environment for the estimation of a scalar water parameter field of interest.

Adaptive trajectory planning has been under extensive investigation in terrestrial robotic networks. Both myopic solutions and non-myopic solutions have been proposed in different contexts (e.g., mapping, physical phenomenon monitoring and field maxima tracking). In myopic solutions, the trajectories (or sampling positions) in the next time step are determined to optimize some predefined performance metric, such as the reduction of the field estimation error [8,9] and the conditional information entropy [10]. Both a single robot [9] and multiple robots [8,10] have been considered in existing solutions. In non-myopic solutions, the trajectories are determined to optimize a long-term performance. Specifically, the trajectory planning is often formulated as a partially observable MDP, in which the state is formed by the robot status and the collected field knowledge, and the action corresponds to the waypoints to travel or parameterized continuous trajectories [11,12,13,14]. The partially observable Markov decision process is typically solved via Bayesian optimization methods and Monte Carlo tree search [11,13,14]. Due to the high computational complexity of Bayesian optimization and Monte Carlo tree search, existing solutions mainly consider a single robot. For multiple robots, the objective function can be designed to have a certain structure (e.g., local submodular) to make the problem computationally tractable [12]. In both myopic and non-myopic solutions, the field is typically modeled as a Gaussian process, and the field covariance function is assumed known a priori, which can be estimated based on historical measurements.

Relative to terrestrial robotic networks, studies on adaptive trajectory planning of underwater AUVs have been very limited. Existing solutions have been developed for feature tracking (e.g., tracking of thermal fronts) at relatively small spatial scales using gradient climbing strategies and for coverage of a field of interest at large spatial scales. This work focuses on the latter applications, such as mapping of the temperature, salinity, flow or biological variables in a two- or three-dimensional water region. To achieve synoptic coverage of the field of interest, coordination among the AUVs via wireless communications is critical to keep the AUVs appropriately distributed in space according to the field spatial variability [4]. Thus far, satellite links are the most common solution for information exchange between an AUV and the control center when the AUV surfaces every few hours (e.g., 2 h). Such a large communication latency prevents timely uploading of data from AUVs and timely update of AUV trajectories, especially in the presence of strong currents.

For the estimation of an unknown water field, due to the communication constraint, existing works have mainly focused on offline AUV trajectory planning. Specifically, the unknown field is typically represented as an uncertainty field described by a Gaussian process with known covariance, and the AUV trajectories are determined to maximize the uncertainty reduction (i.e., the collected information) subject to the constraints on, e.g., the primary motion, anti-curling, vicinity, communications and obstacle avoidance. The AUV trajectory is represented by a series of waypoints on a discrete grid in the region of interest [5,15,16] or parameterized by a restricted number of parameters [4]. For a single AUV, the branch and bound algorithm [5], the recursive greedy algorithm [15] and the sampling-based redundant roadmap method [16] have been applied to find the near-optimal solution. In the presence of multiple AUVs, the AUV trajectories can be jointly computed prior to the deployment by the control center [5].

Although with a human in the loop, the trajectory of an AUV can be adjusted during its deployment via satellite links once it surfaces [4], research on the machinery and online adaptation of AUV trajectories to maximize the collected information has been limited. Two existing myopic solutions to adaptive AUV trajectory planning are described in the following. In References [17,18], to prevent the existence of a single point of failure and to achieve the scalability to the number of AUVs, a decentralized strategy for multi-AUV sampling and patrolling is developed. The region of interest is dynamically partitioned into multiple Voronoi cells according to the AUV locations. Based on the samples collected by all or neighboring AUVs in the past, each AUV computes the next visiting point within its own Voronoi cell in a myopic way to maximize the amount of information to be collected. The above operation requires information exchange among the AUVs. Field experimental results were presented with two AUVs exchanging information via acoustic communications [19] and three surface vehicles exchanging information via radio frequency links [17]. In the above works, the unknown field is modeled as a Gaussian process with known covariance function. Such a priori field knowledge, however, could be difficult to obtain in practice, particularly in the presence of large field dynamics.

**Figure 1:**
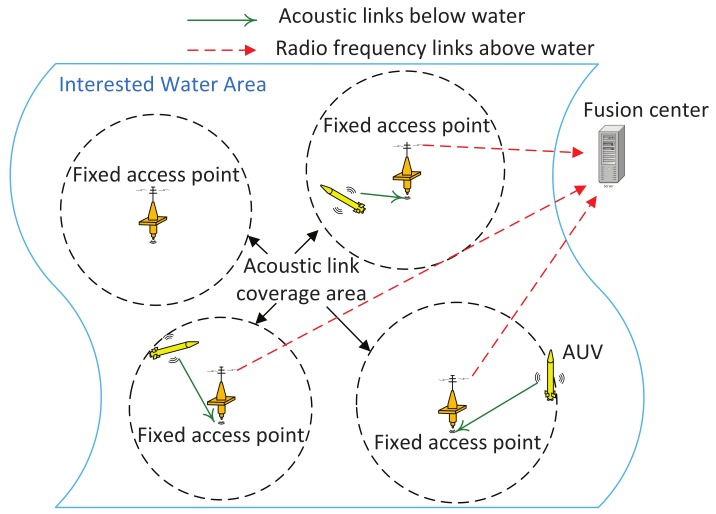
An illustration of the system layout with three autonomous underwater vehicles (AUVs) and four access points.

This work studies the non-myopic adaptive trajectory planning of multiple AUVs in the under-ice environment for the estimation of a water parameter field of interest. In particular, we consider a centralized system as illustrated in Figure 1, where the fixed access points on the ice layer serve as gateways for communications between the AUVs and a remote data fusion center. The AUV trajectories are determined by the fusion center on a time epoch-by-epoch basis based on the samples collected in the past epochs. Different from the open-water AUV network in which the AUVs can access satellite links wherever they surface, the under-ice AUVs can only communicate with the fusion center when they are within the communication range of an access point.

In this work, the water parameter field of interest is modeled as a Gaussian process with an unknown covariance function specified by hyper-parameters [20]. At the end of each epoch, the field samples collected by the AUVs are relayed via the access points to the fusion center where the field hyper-parameters are estimated via the maximum likelihood method [20], and the posterior field distribution and the field uncertainty are computed via the Gaussian process regression [21]. The AUV trajectories in the next epoch will then be determined by the fusion center based on the current system state including the current positions of all the AUVs and the field knowledge, with the aim of maximizing a long-term system reward that is defined based on the field uncertainty reduction and the AUV mobility cost. The AUV trajectories are expected to satisfy several practical constraints, including the kinematics constraint, the constraint on communications and the constraint of being within the area of interest.

The adaptive trajectory planning problem is formulated as a MDP [22] with a constrained continuous action space. A reinforcement learning-based method is designed for online learning of the optimal action, i.e., the trajectories of all the AUVs, which satisfies the constraints. The knowledge for determining the optimal trajectories in each epoch is first obtained by transferring the historical knowledge of determining the trajectories in the previous epoch and then is further adjusted based on the newly-collected reward. The proposed reinforcement learning-based trajectory planning algorithm is validated using simulated two-dimensional (2D) fields. The simulation results show that the proposed algorithm achieves performance similar to a benchmark method that assumes perfect knowledge of the field hyper-parameters.

The main contributions of this work are summarized in the following.

- The developed algorithm is non-myopic and for multiple AUVs, while existing works consider either non-myopic planning for a single vehicle [11,12,13,14] or myopic planning for multiple vehicles [8,10,17,18]. To tackle the high computational cost of non-myopic multi-vehicle planning, instead of using Monte Carlo tree search, we employ a learning algorithm which can be implemented via parallel computation. To further speed up the convergence of the planning algorithm, the decision-making strategy is adjusted on the fly by transferring the knowledge learned in previous epochs.
- By introducing a deep deterministic policy gradient (DDPG) method, the developed algorithm allows adaptive trajectory planning in a continuous action space, while many existing works consider either a discrete action space or a finite number of pre-determined trajectory patterns [10,12,13,14].
- This work performs the online learning of the field hyper-parameters according to the maximum likelihood principle, while many existing works assume those parameters known a priori [5,8,15,17].

**Remark** **1.** *The proposed solution for adaptive trajectory planning can be directly implemented on AUVs that are equipped with an autonomy package, appropriate sensors and an acoustic communication unit. Specifically, with the support of wireless communications between AUVs and a remote fusion center, the field samples collected by AUVs can be sent periodically to the fusion center for centralized processing and planning for the AUV trajectories in the near future. To ensure the communication reliability, both forward error control via error correction coding and backward error control via automatic repeat request can be applied [23]. Different from mission adaptive systems where the AUV uses an on-board decision-making system for online mission planning [24], the decision-making in this work occurs at the fusion center, which can have access to sufficient computational and storage resources. The AUVs are mainly responsible for navigation according to the determined trajectories by the fusion center, taking field samples around waypoints and sending the field samples to the fusion center via gateways (access points).*

**Remark** **2.** *The proposed solution has several advantages for field missions. First, the proposed solution does not require prior knowledge about the field spatial variability, benefiting from the online estimation of the field spatial correlation parameters (the hyper-parameters). Secondly, the trajectory obtained in the proposed solution is not constrained to a set of predetermined discrete points, rather it can be an arbitrary path in the continuous water region of interest. Thirdly, the proposed trajectory planning algorithm is non-myopic, benefiting from the relatively large computational power of the remote fusion center.*

The rest of this paper is organized as follows. The system model is presented in Section 2. The online adaptive trajectory planning is formulated into an optimization problem in Section 3. A reinforcement learning-based algorithm is developed to solve the optimization problem in Section 4. Evaluation of the proposed algorithm is included in Section 5. Conclusions are drawn in Section 6.

**Notation** **1.** *Bold upper case letters and lower case letters are used to denote matrices and column vectors, respectively. $A^{T}$ denotes the transpose of matrix ***A***. ${\left[a\right]}_{m}$ denotes the m-th element of vector ***a***. For $a={\left[a_{1},a_{2},\cdots ,a_{n}\right]}^{T}$, ${∥a∥}_{2}:={\left(a_{1}^{2}+a_{2}^{2}+\cdots +a_{n}^{2}\right)}^{1/2}$ denotes the Euclidean norm (also known as the two-norm) of ***a***. ${\left[A\right]}_{i,j}$ denotes the (i,j)-th element of matrix ***A***. $A^{-1}$ denotes the inverse matrix of ***A***. det(A) denotes the determinant of matrix ***A***. $I_{N}$ denotes an identity matrix of size N×N. |A| denotes the cardinality of set A.*

## 2. System Model

### 2.1. System Description

The system under consideration consists of multiple AUVs, several fixed access points and a remote fusion center. Denote the AUV index set as M={1,2,…,M}. The AUVs are equipped with sensors and acoustic communication units. They take field measurements at discrete sampling locations as they navigate along their trajectories. A total number of $N_{\mathrm{AP}}$ access points are placed at fixed locations, serving as gateways for communications between AUVs and the fusion center. The acoustic links are used for underwater information exchange between AUVs and access points, and the radio frequency links are used for in-air information exchange between access points and the fusion center. With the access points, the data collected by AUVs can be sent to the fusion center for centralized processing. An illustration of the system layout with three AUVs and four access points is shown in Figure 1.

We consider a water parameter field within an area of interest $X_{\mathrm{area}}\subset R^{\mathrm{Dim}}$, with Dim=2 or 3 denoting the dimension of the area. The water parameter field is represented by z(x), with $x\in X_{\mathrm{area}}$. Within the area $X_{\mathrm{area}}$, a discrete set of target points X is selected based on application requirements. The overall system mission is to minimize the field uncertainty (namely, the estimation inaccuracy) over those target points.

**Figure 2:**
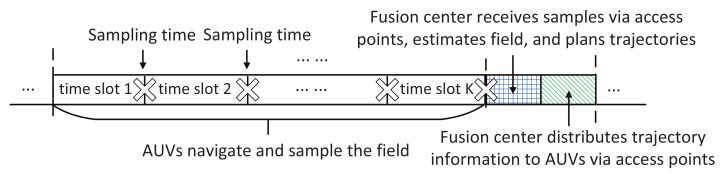
System operation within an epoch. The AUV samples at the end of each time slot. There are *K* time slots within an epoch for AUV navigation and sampling.

The system operates on an epoch-by-epoch basis. An illustration of the system operation within each epoch is shown in Figure 2. The trajectories for all the AUVs in the *ℓ*-th epoch are determined at the end of the (ℓ−1)-th epoch based on the collected field samples. For each AUV, the planned trajectory consists of *K* waypoints. It takes one time slot for the AUV to travel from one waypoint to the next. Each AUV collects field measurements around the waypoints. After the AUV reaches the last waypoint in the current epoch, it transmits the observed data and the corresponding sampling locations to the nearest access point via underwater acoustic links. The access points then relay all the information to the fusion center via radio frequency links. The fusion center estimates the field values at target points in X based on all the observations and updates the estimation of the field spatial correlation parameters. It then determines the trajectories for all the AUVs in the next epoch and transmits via access points the planned trajectories to all the AUVs. At the end of the *ℓ*-th epoch, all the AUVs receive their planned trajectories in the next epoch.

### 2.2. Autonomous Underwater Vehicles Trajectory Modeling

For the *i*-th AUV and the *ℓ*-th epoch, the planned trajectory that consists of *K* waypoints is represented as ${\overset{˘}{X}}_{i}\left(\ell \right):=\left\{{\overset{˘}{x}}_{i,\ell }\left(1\right),{\overset{˘}{x}}_{i,\ell }\left(2\right),\cdots ,{\overset{˘}{x}}_{i,\ell }\left(K\right)\right\}$. The last waypoint in the *ℓ*-th epoch is the AUV starting position in the (ℓ+1)-th epoch, namely ${\overset{˘}{x}}_{i,\ell }\left(K\right)={\overset{˘}{x}}_{i,\ell +1}\left(0\right)$.

Based on the system description, the AUV trajectories need to satisfy some practical constraints. We consider three constraints in the following.

- Kinematics constraint: Due to the limited travel speed of an AUV, the distance between any two consecutive waypoints for each AUV is constrained as:
  (1) $$∥{\overset{˘}{x}}_{i,\ell }\left(k\right)-{\overset{˘}{x}}_{i,\ell }{\left(k+1\right)∥}_{2}\leq \kappa_{\mathrm{up}},\;0\leq k\leq K-1,\;\forall i\in M$$
  where $\kappa_{\mathrm{up}}$ is the maximal distance that an AUV can travel within one time slot.   
- Communication constraint: For each epoch, since the AUV needs to send its field samples to an access point when it arrives at the last waypoint, the AUV should be within the communication range of at least one of the $N_{\mathrm{AP}}$ access points, namely,
  (2) $$∥{\overset{˘}{x}}_{i,\ell }\left(K\right)-x_{\mathrm{AP}}^{\left(j\right)}{∥}_{2}\leq \kappa_{\mathrm{comm}},\;\exists j\in I_{\mathrm{AP}},\;\forall i\in M$$
  where $I_{\mathrm{AP}}:=\left\{1,2,\cdots ,N_{\mathrm{AP}}\right\}$ is the access point index set, $x_{\mathrm{AP}}^{\left(j\right)}$ is the location of the *j*-th access point and $\kappa_{\mathrm{comm}}$ is the communication range that ensures reliable transmission between an access point and an AUV.   
- Sensing area constraint: All the AUVs should stay within the area of interest, namely,
  (3) $${\overset{˘}{x}}_{i,\ell }\left(k\right)\in X_{\mathrm{area}},\;1\leq k\leq K,\;\ell \geq 0,\;\forall i\in M.$$

Due to the water current and obstacles, the AUVs may not arrive at each planned waypoint exactly. In field missions, the “arrival” is defined based on the allowable location discrepancy specified in the mission file. For the *i*-th AUV, we model the *k*-th sampling location within the *ℓ*-th epoch as:

(4) $$x_{i,\ell }\left(k\right)={\overset{˘}{x}}_{i,\ell }\left(k\right)+e_{i,\ell }\left(k\right),$$

where $e_{i,\ell }\left(k\right)\in R^{\mathrm{Dim}}$ is a noise vector, which describes the location inaccuracy, and each of its elements is assumed following an independent and identical uniform distribution U(−ϵ,ϵ) with $\epsilon \ll \kappa_{\mathrm{comm}}$ and $\epsilon \ll \kappa_{\mathrm{up}}$ [25].

### 2.3. Unknown Field Modeling

We model the unknown field z(x) as a Gaussian process with zero mean,

(5) $$z\left(x\right)∼GP\left(0,K(x,x^{\prime })\right),\;\forall x,x^{\prime }\in X_{\mathrm{area}}$$

where $K(x,x^{\prime })$ is the covariance function of the field values at locations **x** and $x^{\prime }$.

There are various types of covariance functions that can be employed to describe the field spatial correlation [20]. In this work, we consider the squared exponential covariance function,

(6) $$K\left(x,x^{\prime }\right)=\sigma_{f}^{2}\exp\left(-{\left(x-x^{\prime }\right)}^{T}\Lambda^{-2}\left(x-x^{\prime }\right)\right),$$

where $\Lambda =\mathrm{diag}(\left[d_{1},\cdots ,d_{\mathrm{Dim}}\right])$ with Dim=2 or 3 being the dimension of the water area and $d_{i}$ being the distance scale that determines the field spatial correlation at two locations and $\sigma_{f}^{2}$ is the signal variance. The matrix **Λ** and $\sigma_{f}^{2}$ are referred to as field hyper-parameters. In this work, we consider the lack of prior knowledge about the field hyper-parameters $\theta_{\mathrm{hyper}}:=\left\{\sigma_{f}^{2},\Lambda \right\}$ and develop a method to estimate the hyper-parameters based on the sequentially-collected field samples by AUVs.

For the *i*-th AUV, the field observation at the *k*-th sampling location in the *ℓ*-th epoch is described as:

(7) $$y_{i,\ell }\left(k\right)=z\left(x_{i,\ell }\left(k\right)\right)+w_{i,\ell }\left(k\right),$$

where $w_{i,\ell }\left(k\right)$ is the observation noise and assumed following a Gaussian distribution $N(0,\sigma_{w}^{2})$.

## 3. Problem Formulation for Adaptive Trajectory Planning

In this section, we develop a mathematical model for the field estimation. The trajectory planning for multiple AUVs is then formulated as a constrained optimization problem, to maximize the field estimation accuracy while minimizing the AUV mobility cost.

### 3.1. Gaussian Process Regression for Field Estimation

Based on the field observations, we use the Gaussian process regression to estimate the field values at target locations and estimate the field hyper-parameters $\theta_{\mathrm{hyper}}$ using the maximum likelihood method.

Stack the observations of all the AUVs in the *ℓ*-th epoch into a vector $y_{\ell }$ of length MK. Stack all the observations up to the *ℓ*-th epoch into a long vector $y_{\mathrm{cum},\ell }:={\left[y_{0}^{T},\cdots ,y_{\ell }^{T}\right]}^{T}$. The observation vector follows a Gaussian distribution,

(8) $$y_{\mathrm{cum},\ell }∼N\left(0,C_{\ell }\right),$$

where $C_{\ell }$ is the covariance matrix. The (i,j)-th element of $C_{\ell }$ is computed as:

(9) $${\left[C_{\ell }\right]}_{i,j}=K\left(x_{i},x_{j}\right)+\delta_{ij}\sigma_{w}^{2},$$

where $x_{i}$ and $x_{j}$ denote the sampling location of the *i*-th element and the *j*-th element in $y_{\mathrm{cum},\ell }$, respectively, and $\delta_{ij}$ denotes the Dirac delta function.

We stack the field values at target locations within the set X into a vector **z** of length |X|. Its posterior distribution based on the observations collected up to the *ℓ*-th epoch is:

(10) $$z∼N(\mu_{\ell },\Sigma_{\ell }),$$

with:

(11) $$\begin{array}{cc} \mu_{\ell } & =C_{z,y_{\mathrm{cum},\ell }}C_{\ell }^{-1}y_{\mathrm{cum},\ell }, \end{array}$$

(12) $$\begin{array}{cc} \Sigma_{\ell } & =C_{z}-C_{z,y_{\mathrm{cum},\ell }}C_{\ell }^{-1}C_{z,y_{\mathrm{cum},\ell }}^{T}, \end{array}$$

where $C_{z}$ is the covariance matrix of **z**, its (i,j)-th element is obtained as ${\left[C_{z}\right]}_{i,j}=K\left(x_{i},x_{j}\right)$ with $x_{i}$ and $x_{j}$ being the location of the *i*-th and the *j*-th element in **z**, respectively, $C_{x,y_{\mathrm{cum},\ell }}$ is the covariance matrix between **z** and $y_{\mathrm{cum},\ell }$ and its (i,j)-th element is obtained as ${\left[C_{x,y_{\mathrm{cum},\ell }}\right]}_{i,j}=K\left(x_{i},x_{j}\right)$ with $x_{i}$ and $x_{j}$ being the location of the *i*-th element in **z** and the *j*-th element in $y_{\mathrm{cum},\ell }$, respectively.

Based on the observation vector $y_{\mathrm{cum},\ell }$, the field hyper-parameters $\theta_{\mathrm{hyper}}=\left\{\sigma_{f}^{2},\Lambda \right\}$ can be estimated by maximizing the log likelihood function [26],

(13) $$\begin{array}{cc} {\hat{\theta }}_{\mathrm{hyper}} & =\max_{\theta_{\mathrm{hyper}}}\ln f\left(y_{\mathrm{cum},\ell };\theta_{\mathrm{hyper}}\right),\\ & =\max_{\theta_{\mathrm{hyper}}}\left\{-\frac{1}{2}y_{\mathrm{cum},\ell }^{T}C_{\ell }^{-1}y_{\mathrm{cum},\ell }-\frac{1}{2}\log\det\left(C_{\ell }\right)\right\}, \end{array}$$

where $f\left(y_{\mathrm{cum},\ell };\theta_{\mathrm{hyper}}\right)=N\left(0,C_{\ell }\right)$ is the probability density function (also known as the likelihood function) of vector $y_{\mathrm{cum},\ell }$ (cf. Equation (8)) and $C_{\ell }$ is related to the hyper-parameters $\theta_{\mathrm{hyper}}$ through Equations (6) and (9). The optimization problem Equation (13) can be solved using a quasi-Newton method, i.e., the L-BFGS-B (limited memory Broyden–Fletcher–Goldfarb–Shannon algorithm for bound constrained optimization) method [27]. The optimization Equation (13) is used to estimate the field spatial correlation parameters, i.e., the field hyper-parameters, based on the collected field measurements at the end of each epoch. The estimated field spatial correlation parameters will then be used for trajectory planning.

### 3.2. Problem Formulation for Optimal Trajectory Planning

The field uncertainty can be computed based on the posterior distribution of **z**. Specifically, we define $u_{\ell }:=\mathrm{diag}\left(\Sigma_{\ell -1}\right)$ to describe the uncertainty of all the target points in X based on the observations up to the (ℓ−1)-th epoch. Denote $p_{\ell }:=\left\{x_{1,\ell }\left(0\right),x_{2,\ell }\left(0\right),\cdots ,x_{M,\ell }\left(0\right)\right\}$ as the locations of all the AUVs at the beginning of the *ℓ*-th epoch. Denote $s\left(\ell \right):=\left\{p_{\ell },u_{\ell }\right\}$ as the system state at the beginning of the *ℓ*-th epoch. Denote a(ℓ) as the action in the *ℓ*-th epoch, which consists of the planned waypoints for all the AUVs in the *ℓ*-th epoch.

For the long-term AUV deployment, the AUV trajectories in all the future time epochs can be optimized to maximize the overall system performance. Mathematically, given the the randomness of the field values (cf. Equations (5) and (7)) and the uncertainty during the AUV navigation (cf. Equation (4)), the desired trajectories for all the AUVs can be determined to maximize the expected total discounted reward,

(14) $$\max_{{\left\{a\left(\ell \right)\right\}}_{\ell =0}^{\infty }}E\left\{\sum_{\ell =0}^{\infty }\gamma^{\ell }R\left(s\left(\ell \right),a\left(\ell \right)\right)\right\},$$

where γ∈(0,1] is a discount factor, R(s(ℓ),a(ℓ)) is an application-dependent reward function and E{·} denotes the statistical expectation of a random variable. Here, the discount factor is introduced to give more preference to the reward in the near future. In this work, the reward function takes into account the field uncertainty reduction, the AUV mobility cost based on the planned trajectories and the constraints in Section 2.2, as defined in the following.

#### 3.2.1. Reward Function

For the ease of exposition, denote the current state as s={p,u} and the planned trajectories as **a**. Denote the next state as $s^{\prime }=\left\{p^{\prime },u^{\prime }\right\}$. The reward, costs and penalties induced by action **a** under the current state **s** and the next state $s^{\prime }$ are in the following.

- Uncertainty reduction reward: Given the system mission objective of minimizing the field uncertainty over target locations in X, the reward associated with the reduction of the field uncertainty by performing action **a** at the system state **s** is defined as:
  (15) $$R_{U}\left(s,a\right):=\frac{\alpha_{R}}{\left|X\right|}\left({\left||u|\right|}_{1}-{\left|\right|u^{\prime }\left|\right|}_{1}\right),$$
  where $\alpha_{R}>0$ is a weighting factor (set as $\alpha_{R}=10$ in the simulation) and ${\left||u|\right|}_{1}$ is the summation of all the elements in **u**, which describes the total estimation error over target locations.   
- Trajectory cost: Notice that the AUV energy consumption increases with the travel distance and the turning angle. The mobility cost associated with action **a** is defined as:
  (16) $$C_{T}\left(a\right):=\alpha_{L}L\left(a\right)+\alpha_{A}A\left(a\right),$$
  where L(a) is the total distance of the planned trajectories based on **a**, A(a) is the total angle that the AUVs travel along the planned trajectories based on **a** and $\alpha_{L}>0$ and $\alpha_{A}>0$ are weighting factors and set as $\alpha_{L}=1\times 10^{-3}$ and $\alpha_{A}=5\times 10^{-2}$ in the simulation.   
- Trajectory constraint penalty: The kinematics constraint in Equation (1) will be addressed in the algorithm design for solving the optimization problem in Equation (14) (to be clear in Section 4.2). The constraints in Equations (2) and (3) are tackled by introducing a penalty term into the objective function, where zero penalty is applied when both constraints are satisfied and an extremely large penalty is incurred when either of the two constraints cannot be satisfied. The constraint penalty is defined as:
  (17) $$C_{P}\left(a\right):=\alpha_{p1}I_{1}+\alpha_{p2}I_{2},$$
  where $\alpha_{p1}$ and $\alpha_{p2}$ are positive values and $I_{1}$ and $I_{2}$ are indication functions for constraint Equations (2) and (3), respectively, which equal one if the corresponding constraint is not satisfied and zero otherwise.

The reward function in Equation (14) is then formulated as:

(18) $$R\left(s,a\right)=R_{U}\left(s,a\right)-C_{T}\left(a\right)-C_{P}\left(a\right).$$

#### 3.2.2. Bellman Optimality Equation

Directly solving the optimization problem Equation (14) is often intractable due to the large action and state spaces. Instead, the Bellman optimality equation is used to obtain the optimal actions [22,28]. Denote $Q^{*}\left(s,a\right)$ as the optimal expected reward by performing action **a** under the current state **s**, which is also called the Q-value function. The Bellman optimality equation for the Q-value function is:

(19) $$Q^{*}\left(s,a\right)=E\left\{R\left(s,a\right)+\gamma \max_{a^{\prime }\in A}Q^{*}\left(s^{\prime },a^{\prime }\right)\right\},$$

where $s^{\prime }$ is the next state, $a^{\prime }$ is the action taken in the next state, A is the action space and the expectation E{·} is performed with respect to the probability distribution of $s^{\prime }$ given **s** and **a**. The optimal action $a^{*}$ under the current state **s** can be obtained by maximizing the optimal expected reward,

(20) $$a^{*}=\arg\max_{a\in A}Q^{*}\left(s,a\right).$$

In practice, the optimal expected reward $Q^{*}\left(s,a\right)$ is not directly available. In the next section, we present a reinforcement learning algorithm to approximate the Q-value function and to generate optimal actions.

## 4. Reinforcement Learning-Based Adaptive Trajectory Planning

The proposed optimization problem Equation (14) is essentially an MDP if the field hyper-parameters are known a priori. It has a continuous action space and a continuous state space, which is generally difficult to solve. In this work, we adopt one type of reinforcement learning mechanism, the actor-critic method, to solve the proposed MDP [28]. Classic reinforcement learning algorithms can be categorized into two types. One type is the actor-based method where an actor is trained to generate optimal actions to maximize the Q-value function directly, while the other type is the critic-based method where a critic is trained to evaluate actions, i.e., to approximate the Q-value function and then select the action that yields the maximal Q-value. The actor-critic method combines the two classic types of reinforcement learning methods to achieve higher learning performance. Specifically, in actor-critic-based algorithms, the actor is trained to generate optimal actions, while the critic is trained to provide action evaluation, which helps the actor to improve its action generation strategy. Among various actor-critic-based algorithms, we employed the deep deterministic policy gradient (DDPG) algorithm [29], which deals with continuous action spaces and has high learning efficiency.

### 4.1. Deep Deterministic Policy Gradient Basics and Design

In the DDPG algorithm, an actor is represented by a neural network, which takes the system state **s** as the input and takes the optimal action **a** under the system state **s** as the output. A critic is also represented by a neural network, which takes the system state **s** and the action **a** as the inputs and takes a Q-value function Q(s,a) as the output. The Q-value Q(s,a) indicates the expected reward after taking action **a** under the system state **s**. In the learning process, the actor network provides the action **a** to be executed under the state **s**. After performing action **a**, the corresponding reward R(s,a) can be obtained. Based on the obtained reward, the weights of the critic network are adjusted to better approximate the Q-value function Q(s,a). Then, the weights of the actor are adjusted using the policy gradient method such that the action obtained by the actor could result in higher expected reward, i.e., higher output of the critic network, which takes the output of the actor network as the input. For more details about the DDPG method, please refer to [29].

**Figure 3:**
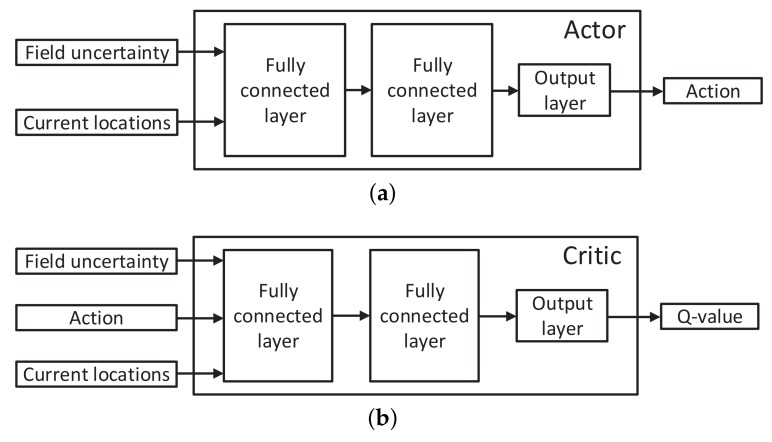
Neural network design in deep deterministic policy gradient (DDPG). (**a**) The forward structure of the actor network; (**b**) the forward structure of the critic network.

A critical issue of the DDPG method is how to design the actor and critic neural networks. In this work, the action for each AUV is parameterized by the moving distance within each time slot along each dimension of the area $X_{\mathrm{area}}$ for *K* time slots in total. The structural design of the actor and the critic is presented as follows. For the actor, as illustrated in Figure 3a, the current field uncertainty and current locations of all the AUVs go through two fully-connected layers with rectified linear units as the activation functions. The output layer takes the summation of the outputs of the second fully-connected layer and uses the tanh activation function to bound the elements of the action to be within [−1,1] (the use of the tanh function will be clear shortly). For the critic, as shown in Figure 3b, the field uncertainty and the current locations and actions of all the AUVs go through two fully-connected layers with rectified linear units as the activation functions. The output layer of the critic is the summation of the outputs of the second fully-connected layer. Consider the online application in this work. The structural design of the actor and critic networks should achieve learning efficiency to balance the system performance and the computational complexity.

In each training iteration, the weights of the actor and the critic networks are updated based on one iteration of the backpropagation algorithm [30].

### 4.2. Training for Actions under Constraints

Consider that the action for each AUV is described by the moving distance within each time slot along each dimension of the area and that each element in the output of the actor network in Figure 3a is constrained within [−1,1] through the employment of the tanh activation function. The kinematics constraint Equation (1) can be met through multiplying each element in the actor output by $\kappa_{\mathrm{up}}/\sqrt{\mathrm{Dim}}$ with Dim=2 or 3 being the dimension of the area, such that the distance that an AUV travels in each time slot is guaranteed to be no greater than $\kappa_{\mathrm{up}}$.

For the constraint Equations (2) and (3), we introduce a technique called experience replay used in the DDPG algorithm [29]. Experience replay is a technique to train the agent by transition samples drawn from a buffer, which consists of historical transitions in previous training experience. Denote the transition from one epoch to the next by a quadruple $\left(s,a,s^{\prime },R\right)$, which consists of the current state **s**, the action **a** performed under the state **s**, the next state $s^{\prime }$ by performing **a** based on **s**, the immediate reward *R* collected by performing **a**. All the historical transitions are stored in a replay buffer denoted by B and will be used for training the actor network and the critic network. Specifically, in each training iteration, the parameters in the actor and critic networks are adjusted by a mini-batch of samples of transitions, which are randomly taken from B. With mini-batch samples, the neural networks can be trained more efficiently compared to the case with one sample per learning iteration. By training based on samples from the replay buffer rather than sequentially-obtained samples, the correlation among training samples can be removed, which improves the convergence performance of the neural networks.

To better learn the actions that satisfy the constraint Equations (2) and (3), we propose a modified DDPG (MDDPG) algorithm where two replay buffers are used for training. Denote $B_{1}$ and $B_{2}$ as two buffers where $B_{1}$ consists of transitions whose actions satisfy the constraint Equations (2) and (3) and $B_{2}$ consists of transitions whose actions do not. By randomly drawing a sufficient amount of transition samples from the buffers $B_{1}$ and $B_{1}$, the actor network and critic network can learn from both “good” and “bad” transition samples with high learning efficiency.

Denote the actor network as μ and the critic network as *Q*. The MDDPG algorithm to obtain the optimal trajectories with the known field hyper-parameters is described in Algorithm 1. In the training process, one training episode refers to a process that begins from the initial state when all the AUVs are at their initial positions (the beginning epoch) and ends at the final state when the whole sampling task is completed (the last epoch). In each epoch, action **a** is randomly adjusted based on the output of the actor according to Algorithm 2. Specifically, an exploration noise is added to generate travel distances, which yield potential higher rewards, and then, the travel distance along a randomly-selected dimension (e.g., longitude, latitude or depth) of the area will be set to zero to introduce trajectories that have less travel angles and also to prevent curling of AUVs. After performing action **a**, the immediate reward *R* and the next state $s^{\prime }$ can be obtained based on Equation (18) and the Gaussian process regression. Instead of learning from the transition quadruple $\left\{s,a,s^{\prime },R\right\}$ immediately, the quadruple is stored in the replay buffers $B_{1}$ or $B_{2}$ based on the condition whether **a** satisfies the constraint Equations (2) and (3) or not. We will train the actor and the critic by a mini-batch of transitions drawn from the buffers $B_{1}$ or $B_{2}$. To ensure that the actor and the critic learn from sufficient samples in both $B_{1}$ and $B_{2}$, the transition samples from $B_{1}$ and $B_{2}$ are drawn, respectively, in two consecutive learning iterations. With the transition samples, the weights of the actor network are updated to minimize the prediction error of the Q-value function, and the weights of the critic network are updated to maximize the Q-value. The stochastic gradient descent method and the target networks are used to update those weights. The target networks are updated by learning the weights of the critic and actor networks with a relatively low learning rate. They provide the action evaluation and generation to update the critic network. The introduction of the target networks improves the learning stability [29]. At the end of the training iteration, the target critic and actor networks are updated.

When the field hyper-parameters are known a priori, the MDDPG algorithm can be used to learn the optimal actions offline. The obtained trajectories can serve as the performance upper bound for the proposed online learning strategy when the hyper-parameters are unknown prior to the system deployment.
**Algorithm 1** Modified deterministic policy gradient (MDPPG) algorithm: $\mathrm{MDDPG}(N_{\mathrm{initial}},N_{\mathrm{episode}},N_{\mathrm{epoch}},N_{\mathrm{batch}},\gamma ,\tau ,\eta ,\beta^{2},Q,W_{Q},\mu ,W_{\mu },Q^{\prime },W_{Q^{\prime }},\mu^{\prime },W_{\mu^{\prime }},\theta_{\mathrm{hyper}},s)$.**Input:** Initial epoch $N_{\mathrm{initial}}$, total training episodes $N_{\mathrm{episode}}$, total epochs in an episode $N_{\mathrm{epoch}}$, mini-batch size $N_{\mathrm{batch}}$, discount factor γ, learning rate of the target networks τ, threshold value η, action adjust variance $\beta^{2}$, the critic network *Q* with its weights $W_{Q}$, the actor network μ with its weights $W_{\mu }$, the target critic network $Q^{\prime }$ with its weights $W_{Q^{\prime }}$, the target actor network $\mu^{\prime }$ with its weights $W_{\mu^{\prime }}$, the field hyper-parameters $\theta_{\mathrm{hyper}}$ and the current system state **s** **Output:** Optimal action set $T_{\mathrm{opt}}$ for future epochs, the critic and actor networks *Q* and μ with weights $W_{Q}$ and $W_{\mu }$, the target critic and actor networks $Q^{\prime }$ and $\mu^{\prime }$ with weights $W_{Q^{\prime }}$ and $W_{\mu^{\prime }}$
1:Initialize replay buffers $B_{1}$ and $B_{2}$. Set $i_{\mathrm{iter}}=0$ and $R_{\mathrm{opt}}=-\infty$2:**for**$\mathrm{episode}=1\;\mathrm{to}\;N_{\mathrm{episode}}$**do**3:    Set $R_{\mathrm{tot}}=0$4:    Set the initial state **s**5:    **for** $\mathrm{epoch}=N_{\mathrm{initial}}\;\mathrm{to}\;N_{\mathrm{epoch}}$ **do**6:        Perform action $a_{\mathrm{epoch}}=\mathrm{RandomAdjust}\left(\mu \left(s\right),\eta ,\beta^{2}\right)$ according to Algorithm 27:        Obtain the immediate reward *R* based on Equation (18), and observe the next state $s^{\prime }$8:        **if** $a_{\mathrm{epoch}}$ satisfies the constraint Equations (2) and (3) **then**9:           Store the transition sample $\left\{s,a_{\mathrm{epoch}},s^{\prime },R\right\}$ into the buffer $B_{1}$10:        **else**11:           Store the transition sample $\left\{s,a_{\mathrm{epoch}},s^{\prime },R\right\}$ into the buffer $B_{2}$12:        **if** $i_{\mathrm{iter}}$ mod 2 **then**13:           Sample a random mini-batch of $N_{\mathrm{batch}}$ transition sample from $B_{1}$14:        **else**15:           Sample a random mini-batch of $N_{\mathrm{batch}}$ transition sample from $B_{2}$16:        For the *i*-th transition sample from the mini-batch $\left\{s_{i},a_{i},s_{i}^{\prime },R_{i}\right\}$, $1\leq i\leq N_{\mathrm{batch}}$, compute $\xi_{i}\leftarrow R_{i}+\gamma Q^{\prime }\left(s_{i}^{\prime },\mu^{\prime }\left(s_{i}^{\prime }\right)\right)$ based on the weights $W_{Q}^{\prime }$ and $W_{\mu }^{\prime }$17:        Update $W_{Q}$ by minimizing the error: $L=\frac{1}{N_{\mathrm{batch}}}\sum_{i}\left|\right|\left(\xi_{i}-Q\left(s_{i},a_{i}\right)\right){\left|\right|}_{2}$ using the backpropagation algorithm [30]18:        Update $W_{\mu }$ by the deterministic policy gradient theorem to maximize $Q(s_{i},\mu \left(s_{i}\right))$ using the backpropagation algorithm [30]19:        Update target networks: $W_{Q^{\prime }}\leftarrow \tau W_{Q}+\left(1-\tau \right)W_{Q^{\prime }}$ and $W_{\mu^{\prime }}\leftarrow \tau W_{\mu }+\left(1-\tau \right)W_{\mu^{\prime }}$20:        $R_{\mathrm{tot}}\leftarrow R_{\mathrm{tot}}+R$, $i_{\mathrm{iter}}\leftarrow i_{\mathrm{iter}}+1$, and $s\leftarrow s^{\prime }$21:    **if** $R_{\mathrm{opt}}<R_{\mathrm{tot}}$ **then**22:        Set $T_{\mathrm{opt}}={\left\{a_{\mathrm{epoch}}\right\}}_{\mathrm{epoch}=N_{\mathrm{initial}}}^{N_{\mathrm{epoch}}}$ and $R_{\mathrm{opt}}\leftarrow R_{\mathrm{tot}}$23:**Return** $\left(T_{\mathrm{opt}},Q,\mu ,W_{Q},W_{\mu },Q^{\prime },\mu^{\prime },W_{Q^{\prime }},W_{\mu^{\prime }}\right)$

**Algorithm 2** Random action adjust: $\mathrm{RandomAdjust}(a,\eta ,\beta^{2})$.**Input:** Action **a**, threshold value η and action adjust variance $\beta^{2}$ **Output:** Adjusted action **a**
1:Draw **w** from a Gaussian distribution $N(0,\beta^{2}I_{\mathrm{Dim}})$ where $I_{\mathrm{Dim}}$ is an identity matrix of size Dim with Dim=2 or 3 being the dimension of the area2:a←a+w and drawn *u* from a uniform distribution U[0,1]3:**if**u<η**then**4:    Uniformly select and set to zero the travel distance along one dimension of the area, and adjust **a** accordingly5:Clip elements in **a** to be within $\left[-\kappa_{\mathrm{up}}/\sqrt{\mathrm{Dim}},\kappa_{\mathrm{up}}/\sqrt{\mathrm{Dim}}\right]$ to meet the kinematics constraint Equation (1).6:**Return a**


### 4.3. Online Learning for Trajectory Planning with Unknown Field Hyper-Parameters

In practice, perfect knowledge of the field hyper-parameters is often unavailable. In this work, we propose an online estimation of the hyper-parameters based on sequentially-collected field samples. An online trajectory planning algorithm, which incorporates the MDDPG algorithm and the online estimation of the field hyper-parameters is described in Algorithm 3. Specifically, after the collection of field samples in each epoch, the unknown field hyper-parameters in the covariance function Equation (6) can be estimated by solving the optimization problem Equation (13) based on all the field observations. After obtaining the estimated hyper-parameters, the previous learned knowledge, including the critic network Q(s,a) with its weights $W_{Q}$, the actor network μ(s) with its weights $W_{\mu }$, the target critic network $Q^{\prime }\left(s,a\right)$ with its weights $W_{Q^{\prime }}$ and the target actor network $\mu^{\prime }\left(s\right)$ with its weights $W_{\mu^{\prime }}$ in the previous epoch, is transferred to the current epoch. The MDDPG algorithm then takes the available knowledge of the actors and the critics and the estimated field hyper-parameters as inputs to learn what will be the optimal trajectories for future epochs. In this way, the optimal trajectories for each epoch can be learned online according to the online estimated field hyper-parameters.
**Algorithm 3** Online trajectory planning algorithm in each epoch.**Input:** Current epoch $N_{\mathrm{curr}}$, total training episodes $N_{\mathrm{episode}}$, total epochs in an episode $N_{\mathrm{epoch}}$, mini-batch size $N_{\mathrm{batch}}$, discount factor γ, learning rate of the target network τ, threshold value η, action adjust variance $\beta^{2}$, the critic network *Q* with its weights $W_{Q}$, the actor network μ with its weights $W_{\mu }$, the target critic network $Q^{\prime }$ with its weights $W_{Q^{\prime }}$ and the target actor network $\mu^{\prime }$ with its weights $W_{\mu^{\prime }}$
1:All autonomous underwater vehicles (AUVs) take samples of the field according to their planned trajectories2:The fusion center receives the field samples from all the AUVs3:The field hyper-parameters $\theta_{\mathrm{hyper}}$ are estimated based on Equation (13)4:The fusion center obtained the updated system state of all the AUVs based on ${\hat{\theta }}_{\mathrm{hyper}}$5:($T_{\mathrm{opt}},Q,\mu ,W_{Q},W_{\mu },Q^{\prime },\mu^{\prime },W_{Q^{\prime }},W_{\mu^{\prime }}$)      $\leftarrow \mathrm{MDDPG}(N_{\mathrm{curr}},N_{\mathrm{episode}},N_{\mathrm{epoch}},N_{\mathrm{batch}},\gamma ,\tau ,\eta ,\beta^{2},Q,W_{Q},\mu ,W_{\mu },Q^{\prime },W_{Q^{\prime }},\mu^{\prime },W_{\mu^{\prime }},\theta_{\mathrm{hyper}},s)$6:Start to perform the action for the next epoch according to $T_{\mathrm{opt}}$


### 4.4. Computational Complexity

The main computational load of the proposed MDDPG algorithm is for neural network training and the hyper-parameter estimation. Denote by *I* the total number of layers in both the actor network and the critic network. Denote by $N_{i}$ the number of nodes within the *i*-th layer. In each epoch, the computational complexity of the MDDPG algorithm is $C_{\mathrm{MDDPG}}=O(N_{\mathrm{episode}}N_{\mathrm{batch}}\left(\sum_{i=1}^{I-1}N_{i+1}N_{i}\right))$. To estimate the hyper-parameters in each epoch, the computational complexity is $C_{\mathrm{est}}=O(\ell^{3})$. Hence, the total computational complexity in each epoch for trajectory planning is $C_{\mathrm{total}}=C_{\mathrm{MDDPG}}+C_{\mathrm{est}}$.

In practice, the computational complexity $C_{\mathrm{total}}$ can be reduced by employing low-complexity algorithms for the matrix multiplication [31] during the neural network training and for the matrix inversion during the Gaussian process regression [32]. Furthermore, the parameters of neural networks $N_{\mathrm{episode}}$, $N_{\mathrm{batch}}$ and $N_{i}$ can be set to relatively small values, which will release the pressure on the computational time. One reason that we can set those parameter to be small is due to the fact that the weights of the neural networks are transferred from epoch to epoch for a warm start. Finally, by using the parallel computation [33], the execution time of the proposed trajectory planning algorithm can be reduced to an acceptable level for practical applications, e.g., less than two minutes.

## 5. Algorithm Evaluation

We consider an under-ice field in a 2D area of interest with size 15 km × 15 km. The target set X consists of 16 × 16 grid points where the latitude and longitude distances between any two consecutive locations are 1 km. The 2D water parameter field is generated based on the circulant embedding method [34] with the field hyper-parameters $\sigma_{f}^{2}=1$ and Λ=diag([0.3,0.3]).

The duration of one time slot is 1000 s (16.7 min), and one epoch consists of three time slots, leading to an epoch duration of 50 min. We consider a total of nine epochs in the sampling process, which yields a deployment time duration of 7.5 h in total. The simulated system consists of four AUVs and four access points. The four access points are located at (4km,4km), (4km,11km), (11km,4km) and (11km,11km), respectively. Those four locations are also the initial deployment sites of the four AUVs. The maximal navigation error is ϵ=5m [35]. The maximal speed of each AUV is 1 m/s [36], and the maximal distance an AUV can travel within one time slot is therefore $\kappa_{\mathrm{up}}=1$ km. The communication range for underwater acoustic links between an AUV and an access point is $\kappa_{\mathrm{comm}}=3.5$ km. The discounted factor is γ=0.99. The weights in the reward function Equation (18) are $\alpha_{R}=10$, $\alpha_{L}=1\times 10^{-3}$, $\alpha_{A}=5\times 10^{-2}$, $\alpha_{p1}=2$ and $\alpha_{p2}=4$.

For both the actor network and the critic network in the proposed MDDPG algorithm, the number of units in the first hidden layer and in the second hidden layer is 400 and 300, respectively. The activation functions of the hidden layers are rectified linear units. The batch normalization is used in the actor network. The learning rate for the actor network and for the critic network is $1\times 10^{-3}$ and $1\times 10^{-4}$, respectively. The learning rate for target networks is $\tau =1\times 10^{-3}$. The mini-batch size for training is $N_{\mathrm{batch}}=10$. The threshold value is η=0.2. The action adjust variance is $\beta^{2}=0.5$ ${\mathrm{km}}^{2}$.

We evaluate the field estimation performance of three schemes.

- Scheme 1: A clairvoyant method that determines the sampling trajectories through the offline MDDPG algorithm based on the perfect knowledge of the field hyper-parameters, according to Algorithm 1;
- Scheme 2: The proposed online reinforcement learning algorithm that determines the sampling trajectories epoch-by-epoch through the MDDPG algorithm where the field hyper-parameters are online estimated in each epoch based on the collected samples, according to Algorithm 3;
- Scheme 3: All the AUVs sample the water parameter field via a random walk. Here, the simulation result to be presented is selected among 10,000 Monte Carlo runs, which yields the maximal total reward.

We take the normalized mean square error (NMSE) as a performance metric for the field estimation, which describes the normalized difference between the true field values and the estimated field values over the target points in X,

(21) $$\mathrm{NMSE}:=\frac{∥z-\hat{z}{∥}_{2}^{2}}{{∥z∥}_{2}^{2}},$$

where **z** is the vector of field values at target points in X and $\hat{z}$ is the estimation based on the Gaussian process regression.

**Figure 4:**
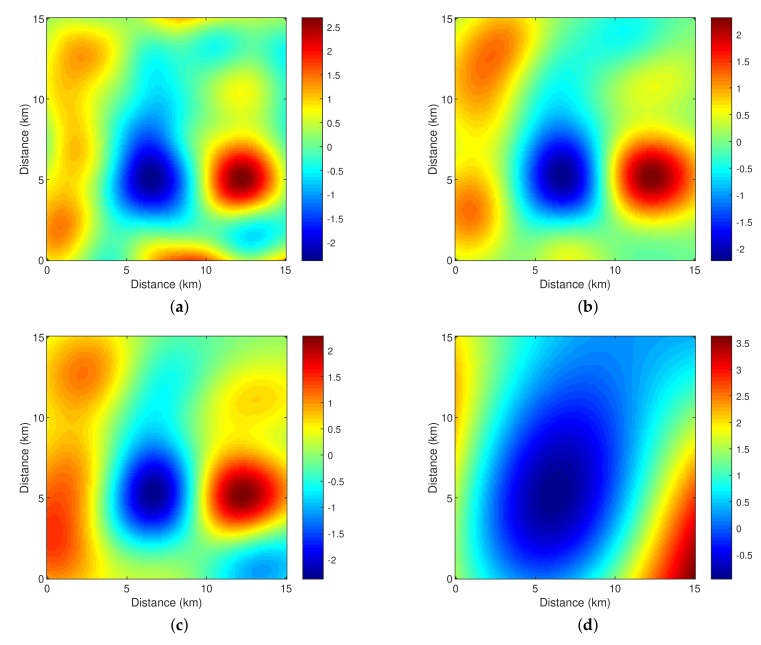
The true field and the estimated fields obtained by the three schemes. (**a**) True field; (**b**) Estimated field by Scheme 1; (**c**) Estimated field by Scheme 2; (**d**) Estimated field by Scheme 3.

**Figure 5:**
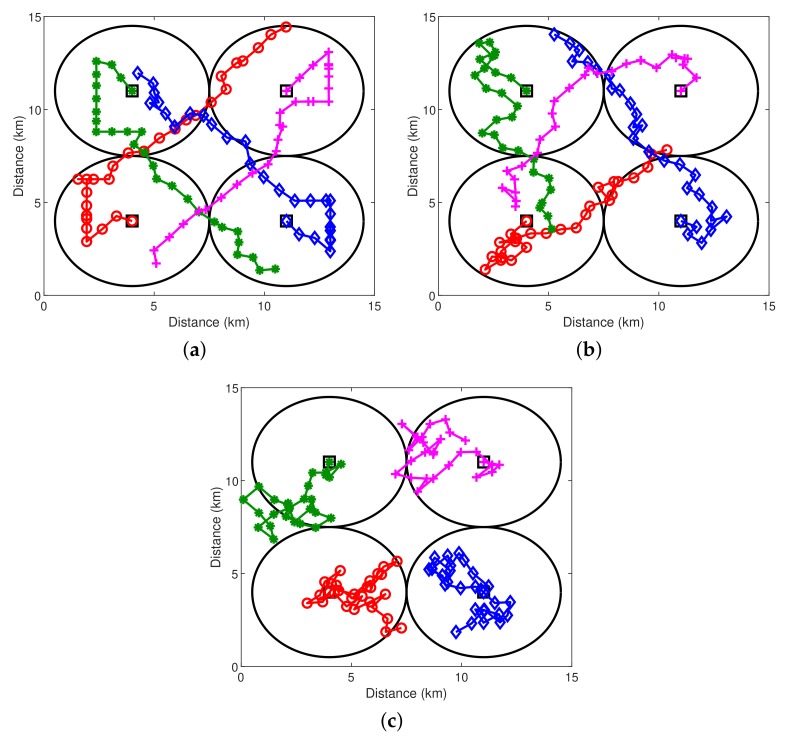
Trajectories of four AUVs obtained by three schemes. The squares indicate the positions of four access points and the initial deployment locations of four AUVs. The circles indicate the acoustic communication coverage of four access points. (**a**) Scheme 1; (**b**) Scheme 2; (**c**) Scheme 3.

Corresponding to the simulated true field in Figure 4a, the trajectories obtained by the three schemes are shown in Figure 5. To explore the area with high uncertainty, the trajectories determined by Scheme 1 spread out more than those of Schemes 2 and 3, which results in the largest sensed area. Without prior knowledge of the field spatial correlation, the sensed area in the early epochs of Scheme 2 is small, primarily due to the inaccurate estimation of the field hyper-parameters based on limited field samples. With more field samples collected and consequently more accurate estimation of the field hyper-parameters, the trajectory pattern obtained by Scheme 2 is similar to the pattern obtained by Scheme 1, which tends to explore the area with high uncertainty.

In Table 1, the three trajectories are compared in the aspects of the AUV total traveled distance, the AUV total traveled angle and the NMSE of field estimation. Scheme 1 achieves the least total traveled distance and the least total traveled angle, while Scheme 2 has a similar total traveled distance, but greater total traveled angle. The performance gap is due to the fact that Scheme 2 does not assume prior knowledge of the field spatial correlation and performs online estimation of the spatial correlation parameters. The total traveled distance and the total traveled angle obtained by Scheme 3 are similar to those of Scheme 2. However, Schemes 1 and 2 achieve much more accurate field estimation (i.e., significantly less NMSEs) than Scheme 3, and a marginal difference of the NMSEs between Schemes 1 and 2 can be observed. The estimated fields by the three schemes are presented in Figure 4. One can see that Schemes 1 and 2 can capture important features of the true field and the estimated field by Scheme 3 is significantly different from the true field.

The above results reveal that although without prior knowledge of the field spatial correlation, the proposed method in this work is able to perform online estimation of the field spatial correlation parameters based on collected field samples and adaptively adjust the trajectories of AUVs while they are on the go. It achieves a performance close to the clairvoyant method assuming perfect knowledge of the field spatial correlation.

Specifically about the proposed method, we further examine the field estimation performance by varying the time epoch duration (namely, the AUV reporting frequency to the fusion center for trajectory adaptation). The number of time slots in each epoch is fixed to be three. The simulation results in Table 2 show that the NMSE of the field estimation decreases as the epoch duration decreases (namely, as the trajectory updating rate increases). Furthermore, the rate of performance improvement becomes less for smaller epoch durations. Consider that such performance improvement is at the cost of more frequent wireless communications and computation at the fusion center for trajectory planning. In real missions, the appropriate choice of epoch duration needs to consider the tradeoff between the field estimation performance and the cost of communications and computation.

## 6. Conclusions

This work studied the online adaptive trajectory planning of multiple AUVs for the water parameter field estimation in the under-ice environment. An online learning-based trajectory planning algorithm was proposed to determine the trajectories of AUVs adaptively. The field of interest was modeled as a Gaussian process with unknown hyper-parameters. The field hyper-parameters and the field posterior distribution were estimated online based on the collected samples. The adaptive trajectory planning problem was formulated as an MDP with the goal of maximizing a long-term reward that is defined based on the field uncertainty reduction and the AUV mobility cost, subject to the kinematics constraint, the communication constraint and the sensing area constraint. A reinforcement learning-based method was designed to solve the above MDP with a constrained action space. The simulation results showed that the proposed reinforcement learning-based adaptive trajectory planning algorithm achieved a performance close to a benchmark method that assumes perfect knowledge of the field hyper-parameters.

## Figures and Tables

**Table 1:** Performance comparison of the three schemes.

	Scheme 1	Scheme 2	Scheme 3
Total traveled distance (km)	74.4	77.9	78.1
Total traveled angle (rad)	76.6	117.4	131.5
Normalized mean square error (NMSE)	0.17	0.26	1.35

**Table 2:** Field estimation performance of Scheme 2 with different values of the epoch duration.

Epoch Duration (minutes)	30	40	50
NMSE	0.22	0.23	0.26
